# Antarctic glacio-eustatic contributions to late Miocene Mediterranean desiccation and reflooding

**DOI:** 10.1038/ncomms9765

**Published:** 2015-11-10

**Authors:** Christian Ohneiser, Fabio Florindo, Paolo Stocchi, Andrew P. Roberts, Robert M. DeConto, David Pollard

**Affiliations:** 1 Department of Geology, University of Otago, PO Box 56, Dunedin 9054, New Zealand; 2 Istituto Nazionale di Geofisica e Vulcanologia, Via di Vigna Murata 605, Rome 00143, Italy; 3 NIOZ Royal Netherlands Institute for Sea Research, Physical Oceanography (FYS), PO Box 59, 1790AB Den Burg, Texel, The Netherlands; 4 Research School of Earth Sciences, The Australian National University, Canberra, Australian Capital Territory 0200, Australia; 5 Department of Geosciences, University of Massachusetts, Amherst, 01003 Massachusetts, USA; 6 Earth and Environmental Systems Institute, Pennsylvania State University, University Park, Pennsylvania 16802, USA

## Abstract

The Messinian Salinity Crisis (MSC) was a marked late Neogene oceanographic event during which the Mediterranean Sea evaporated. Its causes remain unresolved, with tectonic restrictions to the Atlantic Ocean or glacio-eustatic restriction of flow during sea-level lowstands, or a mixture of the two mechanisms, being proposed. Here we present the first direct geological evidence of Antarctic ice-sheet (AIS) expansion at the MSC onset and use a δ^18^O record to model relative sea-level changes. Antarctic sedimentary successions indicate AIS expansion at 6 Ma coincident with major MSC desiccation; relative sea-level modelling indicates a prolonged ∼50 m lowstand at the Strait of Gibraltar, which resulted from AIS expansion and local evaporation of sea water in concert with evaporite precipitation that caused lithospheric deformation. Our results reconcile MSC events and demonstrate that desiccation and refilling were timed by the interplay between glacio-eustatic sea-level variations, glacial isostatic adjustment and mantle deformation in response to changing water and evaporite loads.

## Abstract (web summary)

About 5.6 million years ago the Mediterranean Sea evaporated leaving a 1.5 km deep basin while at the same time Antarctica's ice sheet grew. Here the authors show that growth of Antarctic ice lowered sea-level, which cut off the Atlantic Ocean from the Mediterranean Sea and allowed it to evaporate.

## Graphical abstract

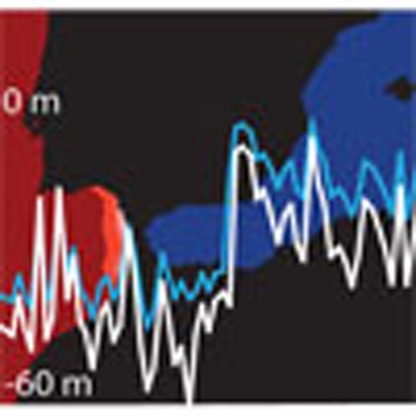

The Messinian Salinity Crisis (MSC)^1^,^2^,^3^,^4^,^5^,^6^,^7^,^8^,^9^ was identified through discovery of thick (up to 3,500 m) evaporitic sediments in the Mediterranean Sea^1^. At the peak of the MSC, the Mediterranean was a series of deep basins^10^ fed only by rivers from Europe and Africa. The ‘lower evaporites' have a basal age of **∼**5.96 Ma, which marks the onset of Mediterranean desiccation at the start of the event. Synchronous brine pool formation across all basins began between 5.75 and 5.60 Ma, culminating in almost complete desiccation by 5.532 Ma (ref. 6). The MSC ended abruptly at 5.33 Ma with catastrophic failure of the Camarinal Sill^9^, which separated the Mediterranean Sea from the Atlantic Ocean. The initial trigger and subsequent evolution of the MSC remain vigorously debated. The modern configuration of the Mediterranean region resulted from relative motions of the African, Arabian and Eurasian plates^11^, and early arguments focused on tectonic restriction between the Mediterranean Sea and the Atlantic Ocean^1^.

Development of deep-sea benthic δ^18^O records suggested that the MSC was influenced strongly by glacioeustasy^4^; however, glacioeustasy was difficult to demonstrate because of poor correlations between key MSC events, δ^18^O records and a lack of direct evidence from Antarctica demonstrating in-phase glacial variability. Previous studies of Antarctic margin successions and glacio-hydro-isostatic adjustment (GIA) modelling^12^ revealed uneven local and global sea-level responses to Antarctic ice-volume changes. Linking the MSC with ice- and water-load-induced variations of relative sea-level (r.s.l.) demands self-consistent modelling^13^,^14^ of solid Earth deformations and concomitant gravitational perturbations that accompany and follow ice-sheet fluctuations. Here we combine geological evidence from the Southern Ocean and Antarctic margin with GIA simulations to show how Antarctic ice-sheet (AIS) evolution, combined with the isostatic response of Gibraltar Strait to water evaporation and salt precipitation, initiated and terminated the MSC.

## Results

### Drill core evidence for ice-sheet growth

We reviewed 60 Southern Ocean and Antarctic margin sedimentary successions to reconstruct AIS evolution during the MSC. Most sites reviewed were not included because they either did not recover MSC-aged sediments or because they had insufficient chronostratigraphic control. We identified four drill sites that contain sediments indicative of ice-sheet expansion during the MSC.

**Figure 1:**
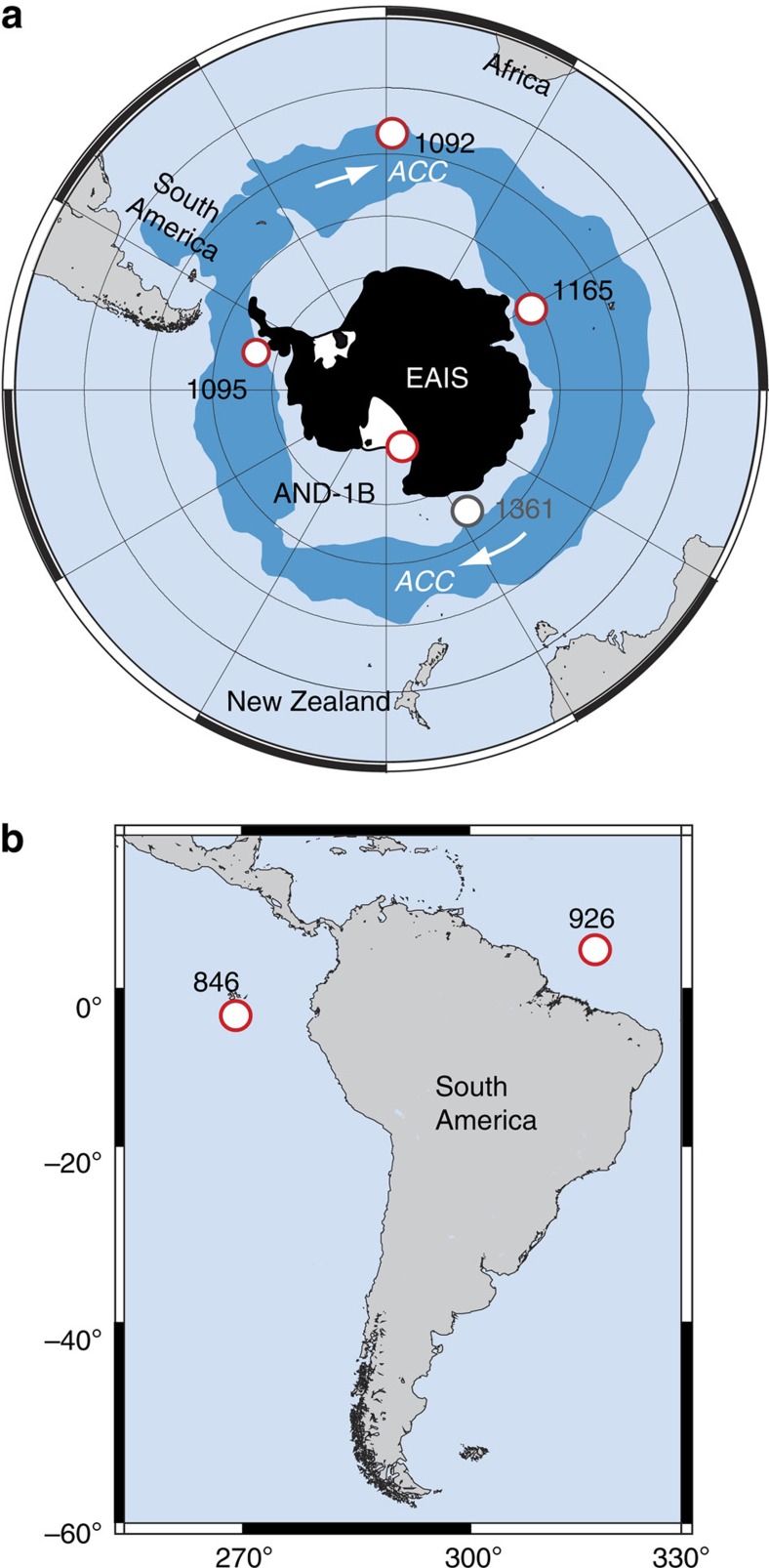
Maps of core locations. Locations of ODP sites 1092, 1095 and 1165 and the AND-1B succession discussed in this study (**a**). ODP sites are situated in deep water and, therefore, track the strength of deep-ocean circulation in response to ice-sheet growth. The AND-1B succession is on the continental shelf and was directly influenced by expanding ice sheets that eroded sediments from the shelf and resulted in glacial erosion surfaces in the succession. A continuous succession spanning the Messinian was recovered at site 1361, but there is no evidence of a significant change in sedimentation or ocean circulation at this site. (**b**) Location map of ODP sites 846 and 926 from which deep-sea δ^18^O records are derived.

Ocean Drilling Program (ODP) sites 1092, 1095 and 1165 (Fig. 1a) were all recovered in relatively deep water (1,000's of metres) and provide a sedimentary archive of Antarctic Circumpolar Current (ACC) evolution in response to AIS growth and decay. Antarctic margin core AND-1B contains a direct record of ice sheet advance and retreat across the continental shelf. Integrated ODP site 1361 recovered a continuous succession spanning the MSC offshore of the Wilkes Land margin in 3,466 m of water. However, no evidence exists at this site for a significant change in sedimentation or ocean circulation, which indicates that this site may not have been sensitive to ice-volume changes during the MSC^15^.

**Figure 2:**
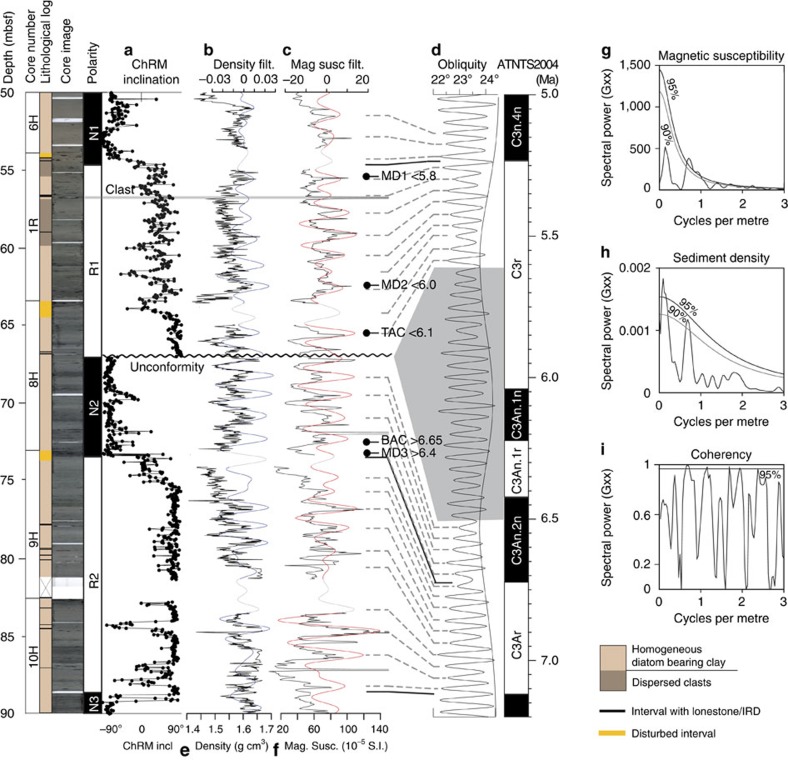
Summary of ODP site 1165 lithology and palaeomagnetic data. ODP site 1165 succession from Prydz Bay, Antarctica, located on the Antarctic Divergence, which comprises a series of gyres at the boundary between the Antarctic Circumpolar Current (ACC) and the Polar Current. The Polar Current and ACC are major surface current systems that extend into Antarctic deep water, and their variable interactions partly control sediment distribution across the continental margin. The ODP site 1165 record between 50 and 90 metres below seafloor (m.b.s.f.). (**a**) Correlation of the magnetostratigraphy (black (grey) data points are from Hole 1165B (Hole 1165C)) with the geomagnetic polarity timescale (ATNTS2012; ref. 19) as guided by five biostratigraphic constraints (markers adjacent to magnetic susceptibility data). (**b**) Glacial/interglacial cycles as expressed in sediment density (**b**, filtered and **e**, raw), and magnetic susceptibility (**c**, filtered and **f** raw) variations are correlated with (**d**) orbital obliquity^66^. Spectral analyses of (**g**) magnetic susceptibility and (**h**) sediment density indicate statistically significant and (**i**) coherent cycles in both records. An unconformity at a depth of 67 m is recognized from the abrupt polarity change and from missing obliquity cycles and has an estimated duration of 890 kyr with an upper and lower age of 5.61 and 6.50 Ma, respectively. Solid black lines indicate unambiguous correlations of magnetic polarity intervals with the GPTS and dashed lines indicate correlation of magnetic susceptibility and density data with the obliquity record.

Sediments at ODP site 1165 were recovered from a water depth of 3,537 m near the East Antarctic coastline at the boundary between the polar gyres and ACC and, critically, near the mouth of Lambert Glacier, which is the largest East AIS outlet glacier, and experienced a ca. 400-km grounding line migration during the latest Miocene^16^,^17^. Here, we focus on the interval between 50 and 90 m below seafloor (5–7 Ma), which contains a previously poorly constrained unconformity^18^ (Fig. 2).

We developed a precise chronology from a revised magnetobiostratigraphy^18^, which facilitated correlation with the ATNTS2012 geomagnetic polarity timescale^19^. Palaeomagnetic analyses reveal a reliable magnetization that results in a well-defined magnetic polarity record with five polarity intervals. Rock magnetic analyses indicate that a mixture of single-domain and pseudo-single-domain magnetite is responsible for the magnetization, and that diagenetic alteration of the magnetite has not occurred. We use biostratigraphic constraints from shipboard observations of first and last appearance datums (FADs and LADs) of diatoms and radiolaria with updated published calibrations to correlate the magnetostratigraphy with ATNTS2012. Marine diatom (MD) datum MD1 is the LAD of *Nitzschia donahuensis*, which occurs at 56.45 m below seafloor (m.b.s.f.), is calibrated at 5.8 Ma (ref. 20), which results in an unambiguous correlation of the R1-N1 reversal with the C3n.4n-C3r reversal. MD2 is the LAD of *Nitzschia miocenica*, which occurs at 63.59 m.b.s.f. and is calibrated at between 6.0 and 6.2 Ma (ref. 18). Top *Amphymenium challengerae* is the LAD of *A. challengerae*, which occurs at 65.45 m.b.s.f. and is calibrated between 6.1 Ma (ref. 18) and 6.2 Ma (ref. 20). Bottom *A. challengerae* is the FAD of *A. challengerae*, which occurs at 72.87 m.b.s.f. and is calibrated at between 6.65 Ma (ref. 18) and 6.8 Ma (ref. 20). MD3 is the FAD of *Thalassiosira miocenica*, which occurs at 73.00 m.b.s.f. and has multiple calibrations of 6.4 Ma (ref. 18), 5.91 Ma (ref. 20) and 6.25–8.3 Ma (ref. 21). For MD3, we use the original calibration of 6.4 Ma (ref. 18), because it follows the downward progression of FADs and LADs most closely. This results in a correlation of the N3-R2-N2 sequence with C3Bn-C3Ar-C3An.2n.

Below the recorded part of Chron C3r, at 67.02 m.b.s.f., a sharp magnetic polarity transition marks a disconformity (Fig. 2a). We determine precisely the upper and lower ages of the disconformity from time-series analysis and bandpass filtering of orbitally paced sediment density and susceptibility cycles (Fig. 2e,f). Sediment density and susceptibility cycles are driven by alternations of biogenic-rich versus terrigenous dominated sediment that are inferred to correspond to alternating warm and productive periods versus colder periods with greater ice volume^22^. Spectral analyses (Fig. 2g,h,i) reveal a dominant wavelength of **∼**0.77 cycles per metre (above the 95% confidence limit for magnetic susceptibility and above 90% for sediment density). Spectral power is greater in the magnetic susceptibility data probably because it is more sensitive to the terrigenous-to-biogenic ratio in sediments; therefore, magnetic susceptibility data were bandpass filtered to isolate the orbital signal (Fig. 2b,c). In total, 27 obliquity-paced glacial–interglacial cycles were identified and correlated one for one with the orbital timescale. The correlation indicates a break in deposition or removal of 890 kyr of sediment between 5.61 and 6.5 Ma. The disconformity probably resulted from current winnowing and non-deposition by erosive bottom currents during AIS expansion^23^. The base of the unconformity (6.5 Ma) marks the downward limit of erosion, not the onset of erosion or ice expansion. Our age model indicates that sedimentation resumed at 5.61 Ma, coincident with the warm, interglacial stage TG15 following a reduction in ocean current speed and ice volume.

**Figure 3:**
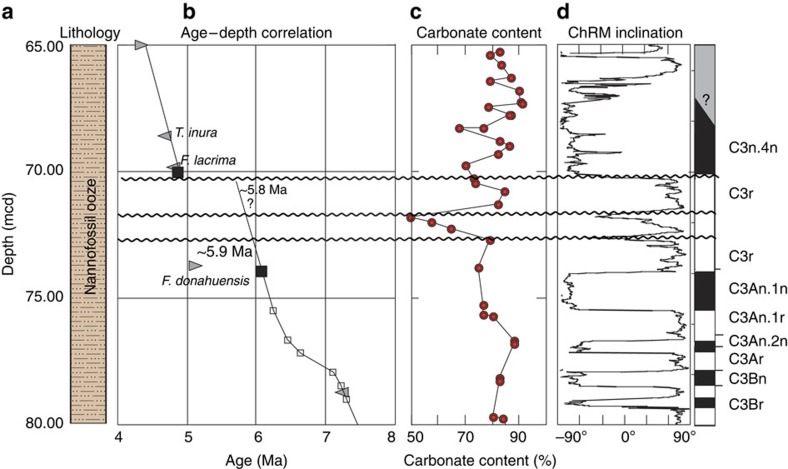
Summary of ODP site 1092 lithology with carbonate content and palaeomagnetic data. ODP site 1092 succession from the South Atlantic Ocean. Sediments comprise (**a**) fine-grained nannofossil ooze and sedimentological analyses indicate an abrupt decrease in (**c**) carbonate content between ca. 72.5 and 71.5 m. (**d**) ChRM inclinations indicate that these intervals have unstable magnetization, and correlation with the ATNTS2012 timescale^19^ indicates that (**b**) one or more unconformities must occur in the succession. The youngest possible upper age for the uppermost unconformity in our age model is 5.8 Ma.

ODP site 1092 comprises a succession dominated by biogenic carbonate (Fig. 3). We refined the age of a poorly constrained unconformity^24^,^25^ using shipboard diatom abundance counts^26^, improved diatom bioevent calibrations^21^ and a reassessment of magnetostratigraphic data^25^. We focus on an interval above the C3An.1n-C3r reversal boundary at 74 metres composite depth (m.c.d.). We used updated LAD and FAD calibrations to assign the normal polarity interval above 70.5 m to chron C3n.4n (Fig. 3d) and to correlate the record with the ATNTS2012 timescale^19^. This correlation is constrained by the unambiguous FADs of *Fragilariopsis lacrima* (69.81 m.c.d.) and *Thalassiosira inura* (68.61 m.c.d.), which have age calibrations of 4.73 and 4.74 Ma, respectively. We use a combination of geomagnetic reversals below the unconformity^25^ and bioevents higher in the succession^26^ and obtained a smooth average sedimentation rate that indicates the presence of one or several unconformities between 72.5 and 70.5 m.c.d. (Fig. 3). The palaeomagnetic inclination data in this interval also indicate at least two intervals with inconsistent palaeomagnetic directions and decreased carbonate content that likely indicate the presence of an unconformity caused by strong, corrosive bottom currents. Our best estimate for the interval that contains these unconformities suggests a basal age of between 5.9 and 5.8 Ma and an upper age of 5 Ma. Additional, unrecognized unconformities in the interval between 70 and 73 m could be present, because the site is located on a bathymetric rise and would have been exposed to erosive currents.

**Figure 4:**
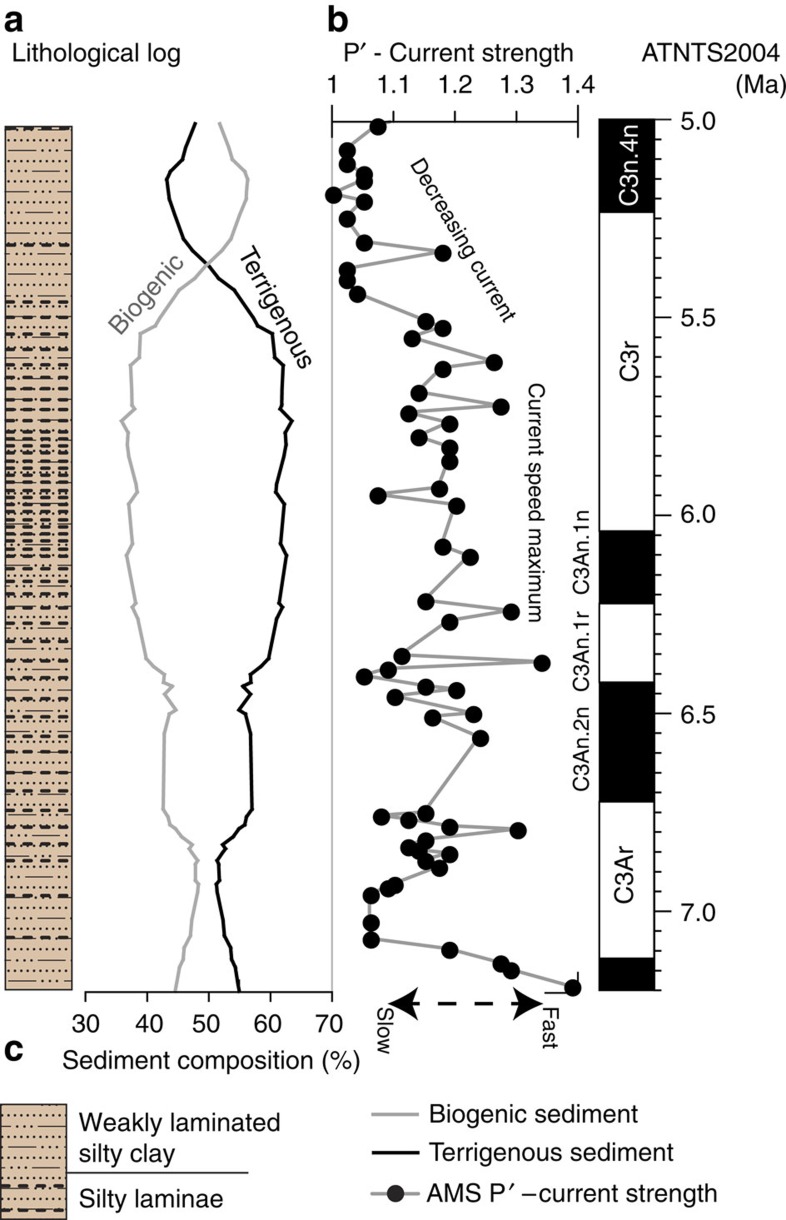
Summary of ODP site 1095 lithology with AMS palaeocurrent proxy and palaeomagnetic data. ODP site 1095 succession from offshore of the Antarctic Peninsula. Sediments comprise (**a**) weakly laminated silty clays with thin-silt laminae throughout the succession. Sedimentological analyses (**c**) indicate that terrigenous sediments are dominant until ca. 5.5 Ma after which biogenic sediments become more dominant and that coarse-grained laminae reach peak numbers between ca. 6.3 and 5.5 Ma, which indicate a strong erosive current system. (**b**) AMS (degree of magnetic anisotropy, *P*′) analyses are indicative of grain alignment in response to ancient current strength with strong grain alignment until ca. 5.5 Ma, which indicate strong, deep currents. AMS data, a decrease in grain size and fewer silt laminae indicate weaker circulation after 5.5 Ma.

Antarctic Peninsula ODP site 1095 contains a continuous succession recovered from a depth of 3,840 m that spans the MSC with a reliable, well-defined chronology^27^ and no evidence for a significant unconformity (Fig. 4). However, sedimentological analyses (Fig. 4a,c) and anisotropy of magnetic susceptibility (AMS) data shed light on changes in ACC strength^28^. AMS provides a measure of fabric strength in the sediment (*P*′, Fig. 4b) where strong fabrics indicate well-aligned grains and an inferred increased current strength. Sedimentological analyses and core logs indicate that the sediments comprise repetitively bedded, weakly laminated silty clays with prominent, graded silt laminae that are interpreted to have been deposited under stronger current regimes. Silty laminae increase in number up-core, which indicates stronger circulation^29^; sedimentary and AMS analyses indicate increased delivery of terrigenous material and an overall stronger grain alignment at **∼**6.3 and **∼**5.6 Ma (ref. 28), which indicate greater current speed likely in response to greater ice volume. Circulation was strongest between 6 and 5.6 Ma with weakening currents after 5.6 Ma and minimum circulation at ca. 5.3 Ma coincident with reduced grain size^29^ and increased biogenic productivity that is interpreted to indicate reduced ice volume^28^.

**Figure 5:**
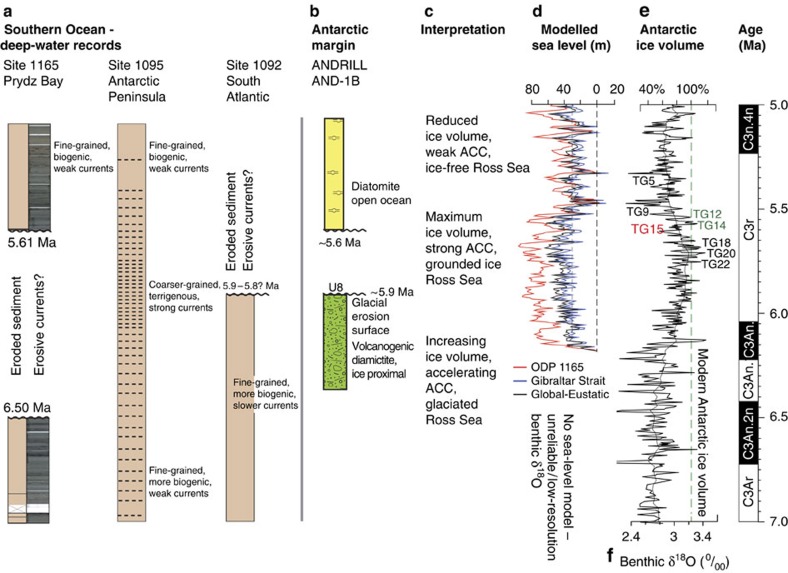
Regional event summary from drill cores with modelled sea-level and Antarctic ice-volume benthic δ^18^O. Regional event summary compiled using (**a**) Southern Ocean ODP successions, (**b**) the Antarctic Margin AND-1B record and ice-volume/ocean current interpretations derived from sedimentary successions. (**c**) Interpretation of Antarctic climate events. (**d**) GIA only modelled r.s.l. at ODP site 1165 (red), at the Strait of Gibraltar (blue) and global eustatic sea level (black). (**e**,**f**) The δ^18^O record^38^,^39^, marine isotope stages and isotope-to-ice volume calibration used for sea-level modelling.

The AND-1B succession (Fig. 5b) is the most ice-sheet-proximal record recovered from the Antarctic margin and contains a record of AIS advance and retreat history that spans the late Miocene to Holocene^30^. The succession comprises massive to stratified diamictites that represent grounded ice or ice-proximal conditions, muddy units that represent ice distal conditions and diatomite intervals that represent deposition in open-marine conditions, in some cases with minimal sea ice^31^,^32^,^33^. Transitions from warm, low-ice-volume conditions to cool, high-ice-volume conditions are typically separated by surfaces where the ice sheet advanced over the drill site and eroded sediment. The succession contains a glacial erosion surface ‘U8' at 596.35 m.b.s.f., which has an estimated age range between 5.90 and 5.60 Ma (Fig. 5b)^34^. Above unconformity U8, a switch from glacially dominated conditions to open-marine conditions is recognized along with the appearance of *Shinodiscus tetraoestrupii* diatoms that are indicative of warm surface conditions (7–10 °C)^32^ and likely much lower ice volume.

### Reconstructing AIS history and sea level

AIS variations exerted a primary control on global sea level on short geological timescales from **∼**34 Ma (ref. 35) until expansion of large northern hemisphere ice sheets after **∼**2.7 Ma (ref. 36). The presence of large northern hemisphere ice sheets will have likely amplified global eustatic sea-level variations^37^. AIS history was largely inferred from benthic δ^18^O records until recovery of well-dated Antarctic margin successions^30^. These ice-sheet-proximal geological records and subsequent modelling studies reveal that ice sheets grew slowly and retreated rapidly during the late Neogene^17^,^30^. To determine realistically how the sea level evolved, we conducted numerical GIA simulations by means of the sea-level equation (SLE)^13^,^14^. Solving the SLE requires a solid Earth model for crustal and gravitational response^13^,^14^ and an ice-sheet chronology as a forcing function, which we generated by scaling present-day AIS thickness using a δ^18^O-based ice volume curve^38^.

### AIS volume and thickness reconstructions

For the time interval under consideration (6–5 Ma), Antarctic ice-sheet thickness variations are unavailable from continuous global circulation model studies. Therefore, we reconstructed AIS volume (Fig. 5e) using benthic δ^18^O records from ODP site 926 (Ceara Rise, 3,598 m water depth, Fig. 1b, ref. 39) between 7 and 6.138 Ma and from ODP site 846 (south of the Galapagos Islands, 3,296 m water depth, Fig. 1b, ref. 38) for the interval between 6.137 and 5 Ma (Fig. 5). We estimate AIS volume using the most conservative approach possible by assuming the modern-day AIS isotopic weight of **∼**−53.2‰, total melted water volume of 22.279 × 10^6^ km^3^ and a 1,335 × 10^6^ km^3^ global ocean volume^40^. Therefore, a total loss of the modern AIS would result in a **∼** 0.91‰ inflection of the deep-sea δ^18^O record. Accordingly, we estimate a **∼**58% reduction of Antarctic ice volume during oxygen isotope stage TG5 from a **∼**0.53‰ inflection in the δ^18^O record. However, to test for different glacial to interglacial temperature contributions to the deep-sea δ^18^O record, we developed two additional ice-volume records that correct for a ca. 20% and 30% temperature contribution (2 and 3 °C), respectively,^41^ in agreement with Mg/Ca records that indicate a ca. 2 °C temperature variation during the Miocene^42^. We tested several other methods to convert benthic δ^18^O to ice volume, including using isotopically heavier ice and the relatively well-understood Pleistocene isotope to sea-level calibration of 0.01‰ m^−1^. The calculated ice-volume fluctuations are amplified, which results in too many intervals with negative ice volume (15% of the record). The rationale for converting with isotopically heavier ice is plausible because it is likely that Miocene ice sheets were warmer and, therefore, isotopically heavier than the modern AIS (some studies indicate that the Oligocene AIS likely had an isotopic weight of **∼**−35‰ (refs 43, 44)). However, we used a more conservative modern **∼**−47‰ isotopic weight, which results in more reasonable ice-volume changes. We also tested the Pleistocene to early Pliocene benthic δ^18^O to sea-level calibration of 0.01‰ m^−1^ (refs 45, 46), which removes the temperature contribution from the record. We computed ice volumes from the sea-level curve by assuming a modern Antarctic sea-level contribution of 56.6 m (ref. 40), which resulted in unrealistically large ice-volume changes. Including a potential sea-level contribution of 7.3 m from the Greenland Ice Sheet (GIS) produced negligible changes to the ice-volume record. Changes in GIS size and other sources of unidentified northern hemisphere ice would have contributed to variations in the δ^18^O record; however, the modern GIS comprises only **∼**7% of the global ice volume, therefore, the majority of δ^18^O variations can be attributed to AIS changes. We converted the high-resolution, orbitally tuned δ^18^O record from ODP site 846 (ref. 38) to ice volume and, accordingly, scaled present-day AIS thickness over time and used this for the GIA simulation.

### GIA and sea-level modelling

We performed a numerical GIA simulation to reconstruct the impact of AIS volume changes on r.s.l. at the Antarctic margin and at Gibraltar. Any ice-sheet fluctuation results in local r.s.l. changes (that is, vertical geoid variations with respect to the deforming solid Earth surface) that stem from a complex interplay between gravitational, rotational and solid Earth deformations in response to redistribution of surface ice- and water loads^47^. Spatial variability of r.s.l. change depends on the distance from the changing ice sheets and on the shape and size of ocean basins^48^. Second, given the viscous behaviour of the solid Earth on geological timescales, ice-induced r.s.l. change varies in time as a function of mantle viscosity. Local r.s.l. change can, therefore, be significantly different from the globally uniform glacio-eustatic sea-level change. Therefore, correlating r.s.l. change at a given location to a specific ice-sheet volume variation requires precise spatio-temporal discretization of the latter (that is, how much ice thickness changed, and where and when this occurred) and a rheological model for the solid Earth response. These two main factors enter into the gravitationally self-consistent SLE whose solution provides the global r.s.l. change. To determine local r.s.l. at a given point, we solved the SLE using the pseudo-spectral method including consistent time-dependent coastline evolution and rotational feedback to meltwater redistribution^49^,^50^,^51^,^52^. We employ a radially stratified, spherically symmetric and rotating Earth model characterized by an upper elastic, 100-km-thick lithosphere, a three-layer Maxwell viscoelastic mantle with an inner mantle viscosity of 5 × 10^21^ Pa s, an outer mantle viscosity of 0.5 × 10^21^ Pa s and an inner inviscid core. Our initial AIS is smaller than at present, so we also modified the present-day initial global topography model ETOPO1 (ref. 53) by melting the excess mass from the present-day AIS and allowing a 50-kyr viscoelastic relaxation via the SLE. We also decreased the depth of the Gibraltar Strait to 30 m below mean sea level^54^,^55^, which is shallower than the modern, deep channel that was eroded at the end of the MSC^9^.

## Discussion

Geological evidence and modelling studies indicate that ice sheets often reach their greatest size immediately before a rapid ice retreat^17^,^30^. Maximum ice volume in Antarctica occurred before 5.61 Ma (Fig. 5e), during δ^18^O stages TG22 through TG18, which was a prolonged period of heavy-δ^18^O values (**∼**5.75 to **∼**5.67 Ma), and inferred 20–30% ice-volume increase^41^.

In the deep-ocean successions assessed here (ODP 1092, 1095 and 1165), we recognize locally increased deep-current velocities after 6 Ma with eroded sediment at ODP sites 1092 and 1165, and sedimentological and AMS evidence at site 1095 for peak circulation between ca. 6.1 and 5.6 Ma (Fig. 4), which likely indicates increased ice volume at this time^23^. The erosion surface in AND-1B also provides direct evidence of ice-sheet expansion during this period where unconformity U8 indicates expansion of an erosive ice sheet in the Ross Sea embayment sometime after 5.95 Ma.

The benthic δ^18^O record provides strong supporting evidence for a period of AIS growth that spanned the duration of the MSC. A period of isotopic enrichment began at ca. 6.2 Ma and continued until ca. 5.75 Ma, which indicates a ca. 50% ice-volume increase that culminated in the large glaciations (TG 22, 20 and 18) during which AIS volume was probably greater than today. Our GIA sea-level simulations indicate substantial sea-level falls with a gradual base-level fall of 50–60 m between 6 and 5.6 Ma at the Gibraltar Strait (Fig. 5d). Sea-level fall at Antarctic proximal ODP site 1165 is more pronounced because of the gravitational pull effect of the ice sheet on the ocean and the growth of a peripheral bulge in response to ice loading^12^. The only sedimentary record from which we can make inferences during the ice-volume maximum at ca. 5.6 Ma is ODP site 1095 where numerous coarse-grained silt laminae dominate the succession and where AMS data indicate maximum current speeds (Figs 4 and 5a). At all other sites, sediments were either not deposited because current speeds were too great or sediments were eroded.

The deglaciation phase began abruptly after ca. 5.6 Ma and isotopically light peaks in the benthic δ^18^O record (TG 9 and 5) indicate rapid deglaciation phases at ca. 5.48 and 5.33 Ma (Fig. 5f). At ODP site 1095, a change to biogenic-dominated, finer-grained sediments and decreased AMS lineation indicates that current speed decreased markedly after 5.6 Ma (Fig. 4). At ODP site 1165 sedimentation resumed, which indicates sufficiently slow circulation to allow sediment deposition, and at Antarctic margin site AND-1B diatomite deposition after 5.6 Ma (ref. 34), which is rich in *S. tetraoestrupii*, indicates ice-free, warm (7–10 °C), open-ocean conditions^32^ and significant ice-sheet reduction possibly coincident with the TG9 isotope event. GIA simulation for this period indicates abrupt sea-level rise coincident with the lightest peaks in the isotope record (Fig. 5d–f). Age model uncertainties in the younger part of the ODP site 1092 record prevent identification of contemporaneous sediments.

**Figure 6:**
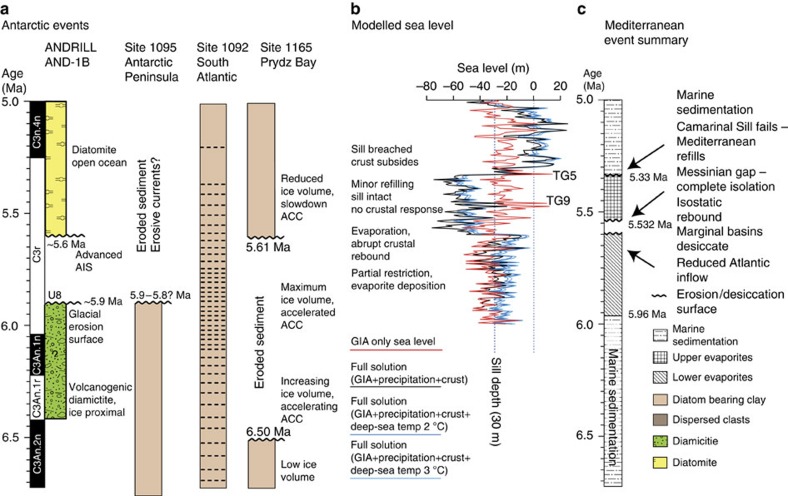
Antarctic and Mediterranean event summary with a modelled sea level at Gibraltar. Chronology of (**a**) Antarctic ice-sheet growth/retreat as interpreted from drill core successions, (**b**) modelled sea level for Gibraltar Strait and for ODP site 1165 from the benthic δ^18^O and ice-volume record and (**c**) Mediterranean desiccation/reflooding events. Sea level at Gibraltar (**b**) is determined from four solutions: a GIA-only solution with no compensation for deep-sea temperature variations (red), a full solution (black) that includes the influence of salt precipitation and the crustal response and two alternative, full simulations with δ^18^O-derived ice-volume reconstructions that compensate for a 2 °C (dark blue) and 3 °C (light blue) glacial–interglacial deep-sea temperature component in the δ^18^O record.

The GIA model indicates a r.s.l. drop at ODP site 1165 that was almost twice as large as the eustatic decrease (Fig. 5d), as a consequence of ice- and water-load-induced uplift of the peripheral forebulge^12^. Conversely, a 5–10 m smaller-than-eustatic r.s.l. drop is predicted at the eastern end of Gibraltar Strait. Here, a gradual, average r.s.l. fall of **∼**40 m occurs between the beginning of the simulation (6.18 Ma) and 5.69 Ma in concert with numerous, short-period sea-level fluctuations above and below the Gibraltar Strait seafloor that coincide with the earliest evaporite accumulation in Mediterranean basins between 5.96 and 5.6 Ma (refs 6, 54, 55, 56, 57, 58), when the Mediterranean could still have been affected by Atlantic water inflow. However, after 5.57 Ma, which coincides with the lowest modelled r.s.l. at Gibraltar (Figs 5d and 6b), the average r.s.l. trend culminates with a rise of **∼**60 m at the two peaks during interglacial TG9 (5.49 Ma). Accordingly, the Mediterranean Sea would have been fully connected to the Atlantic Ocean by 5.53 Ma, which is at odds with the timing of key MSC events, that is, the ‘Messinian gap' during which the Mediterranean Sea was isolated^6^. This discord indicates that the local crustal and geoidal response to Mediterranean desiccation (by evaporation) and to deposition and redistribution of salts, operated in such a way as to counteract the GIA-driven r.s.l. rise between 5.57 and 5.47 Ma. Accordingly, we re-ran our GIA simulation with inclusion of the solid Earth and gravitational response to: (i) deposition of lower evaporites between 5.96 and 5.6 Ma, (ii) rapid drawdown of the Mediterranean sea level because of evaporation to −1,500 m after 5.6 Ma (compensated by **∼**8 m of global sea-level rise), (iii) erosion and re-sedimentation of lower evaporites within the remaining deeper basins and (iv) deposition of upper evaporites in the deeper basins. We applied a weightless vertical barrier at 5.6 Ma to prevent any refilling caused by sea-level highstands and ran the simulation for three ice-volume scenarios (Fig. 6b) that correspond to different deep-sea temperature contributions to the δ^18^O record (see discussion above).

The sea-level curve that results from our reanalysis contains a major change at 5.6 Ma (Fig. 6), where Mediterranean evaporation results in strong uplift at Gibraltar and, therefore, a sudden r.s.l. drop that keeps Atlantic waters 40–50 m below the sill and completely isolates the Mediterranean. However, the two highstands associated with interglacial TG9 reach and overtop the sill (Figs 5d–f and 6) and, without the barrier, would cause Mediterranean refilling. This is consistent with some geological evidence, including isotopic analysis of otoliths^56^, erosion surfaces^57^, coral reefs^58^, reflection seismic data^59^,^60^ and drill core evidence^5^, which indicate limited circulation between the Atlantic and the Mediterranean at ca. 5.46 Ma. In the Sorbas Basin, ^87^Sr/^86^Sr indicates that it may have been connected with the Atlantic during the MSC with ^87^Sr/^86^Sr similar to sea water^61^. However, ^87^Sr/^86^Sr analyses from numerous sites throughout the Mediterranean basin indicate that freshwater input was dominant^61^. In addition, complete Mediterranean refilling at this time as suggested from geological observations in the Sorbas Basin^62^ is not compatible with our simulation, because it would result in crustal subsidence and a return to permanent, fully marine conditions throughout the Mediterranean, which is not supported by geological evidence^6^,^7^,^60^.

The next event capable of overtopping the sill was at 5.33 Ma, in agreement with the geological record and coincident with interglacial TG5 (the Zanclean flooding event^6^). Our evidence indicates that AIS retreat at 5.33 Ma and the associated rapid sea-level rise, in concert with breaching of the barrier at Camarinal Sill, resulted in marked deepening of Gibraltar Straits (Fig. 6) that created a deep channel between the Mediterranean and Atlantic, which has remained open ever since. This study helps to reconcile ice-proximal and far-field sea-level and sedimentation data surrounding an important event in Earth history^60^. We demonstrate that understanding the complex interplay between varied geological processes that contributed to the MSC requires a complex systems approach with dynamic coupling of climatology, oceanography, geodynamics and sedimentology.

## Methods

### Time series analysis

Data from ODP site 1165 from disturbed intervals, significant outliers and from within 8 cm of core ends were removed before spectral analysis and filtering to prevent distortion of the spectral power and filtered data. Spectral and coherency analyses were conducted on magnetic susceptibility and sediment density data (Fig. 2g–i) between 68 (below the unconformity) and 82 m.b.s.f. (the base of core 9H). Spectral analyses were conducted below the unconformity to prevent the phase change from creating artefacts in the spectral power plots. Spectral analyses were performed using the REDFIT^63^ and SPECTRUM^64^ software, where smoothing, Welch-overlapped-segment-averaging with a 50% overlapping window and a Hanning taper were used to define spectral peaks. In all spectral analyses an oversampling factor of 4, a highest frequency factor of 1 and a 0.05 significance level (*λ*) were used. Bandpass filtering was performed using the Analyseries software^65^, where Gaussian filters were centred at 0.77 cycles per metre as determined from spectral analysis with a bandwidth of ±0.25 cycles per metre. Orbital records and insolation for 65°S/N were calculated using Analyseries^65^,^66^.

### Palaeomagnetic and rock magnetic analyses

Palaeomagnetic measurements were made on discrete samples from ODP site 1165 using a 2-G Enterprises superconducting rock magnetometer with in-line alternating field demagnetizing coils. Samples were demagnetized at 10-mT increments to fields of 80 mT, and data were visualized using the PuffinPlot software package^67^. Polarity determinations were made from characteristic remanent magnetization (ChRM) directions using principal component analysis. Low maximum-angular deviation values indicate a low noise level for demagnetization data with vectors that are directed towards the origin of orthogonal vector component plots. A steep drilling-induced overprint is removed at 10 mT, which validates the shipboard magnetostratigraphy constructed from demagnetization data obtained at 20 mT. Demagnetization behaviour indicates a low-coercivity magnetic mineral with linear demagnetization trajectories. To determine more precisely the magnetic mineralogy, first-order reversal curve (FORC) (ref. 68) measurements were made at the Istituto Nazionale di Geofisica e Vulcanologia, Rome, Italy, on a Princeton Measurements Corporation vibrating sample magnetometer (MicroMag 3900). Data were processed using the FORCinel software package^69^ and a smoothing factor of between 5 and 8 was applied to data. FORC diagrams indicate the presence of mixed single-domain, pseudo-single-domain and superparamagnetic magnetite assemblages.

### Crustal and SLE modelling

We solve the SLE to compute the r.s.l. changes that accompany and follow the deposition of salts (lower evaporites first, then upper evaporites) and evaporation of Mediterranean sea water. This is done consistently (in space and time) with the ‘background' GIA signal that is dictated by our AIS chronology (stored as spherical harmonic decomposition of global r.s.l. changes and time-dependent topographies up to degree 256).

Lower evaporite deposition is dated at 5.96 Ma (ref. 6), occurs within predefined boundaries and is consistent with the bathymetry that results from the GIA simulation. We assume an average density of 2,250 kg m^−3^ for the evaporites (see www.simetric.co.uk/si_materials.htm). The resulting r.s.l. changes are overlapped with the GIA signal within the SLE recursive scheme^48^,^49^,^70^ needed to conserve the water mass with the requirement that at any time the sea surface is an equipotential surface of the Earth's gravity field. Accordingly, the bathymetry is updated to account for evaporite deposition.

Following the same procedure, at 5.6 Ma and coincident with a GIA-driven r.s.l. drop, the Mediterranean sea surface is lowered to −1,500 m in agreement with other work^71^ and the equivalent amount of water is moved to the global ocean resulting in **∼**8 m of eustatic rise. Accordingly, two ocean functions now exist: one for the Mediterranean basin and one for the rest of the oceans. However, the sea surface of both ocean functions is an equipotential surface of the Earth's gravity field. Simultaneously, we place a weightless wall of infinite height to prevent any refilling from the Atlantic. This enables us to check whether and when a local r.s.l. rise could refill the Mediterranean basin. We find that the two highstand peaks at TG9 are capable of overtopping the Gibraltar sill. Following the Mediterranean drawdown, we erode the lower evaporites that are now subaerially exposed and place them into the deeper basins. Here we also allow for a final deposition of upper evaporites until 5.33 Ma. By removing the wall at 5.33 Ma we allow sudden, catastrophic refilling of the Mediterranean that results in a marked r.s.l. rise.

### Ice-volume reconstruction

AIS ice-volume calculations assumed a West Antarctic ice volume of 2.9 × 10^6^ km^3^ and an oxygen isotopic weight of −42.5‰, an East Antarctic ice volume of 21.7 × 10^6^ km^3^ and oxygen isotopic weight of −56.5‰ (refs 40, 72), a 10% reduction in ice volume after melt, a global ocean volume of 1,335 × 10^6^ km^3^ (ref. 53) and a modern deep-sea δ^18^O value of 3.38‰.

## Additional information

**How to cite this article:** Ohneiser, C. *et al.* Antarctic glacio-eustatic contributions to late Miocene Mediterranean desiccation and reflooding. *Nat. Commun.* 6:8765 doi: 10.1038/ncomms9765 (2015).
